# Clinical Significance and Prognostic Value of miR-28-5p in Colon Cancer

**DOI:** 10.1155/2020/3159831

**Published:** 2020-05-20

**Authors:** Ji-lin Li, Ke-zhi Li, Ming-zhi Xie, Yan-ping Tang, Yin-lin Tang, Bangli Hu

**Affiliations:** ^1^Department of Research, Guangxi Medical University Cancer Hospital, Nanning 530021, China; ^2^Department of Chemotherapy, Guangxi Medical University Cancer Hospital, Nanning 530021, China; ^3^Clinical Laboratory, Maternal and Child Health Hospital of Guangxi, 530003 Nanning, Guangxi, China

## Abstract

**Background:**

The association of miR-28-5p with colon cancer remains to be elucidated. This study aimed to determine the clinical significance and prognostic value of miR-28-5p in colon cancer.

**Methods:**

We retrospectively analyzed the data of miR-28-5p in colon adenocarcinoma data from The Cancer Genome Atlas (TCGA) and Gene Expression Omnibus (GEO), and the data was divided into cancer group and normal group, respectively. Forty colon cancer tissues and adjacent normal tissues were collected and tested by qRT-PCR methods. The difference of the miR-28-5p expression between colon cancer and normal tissues was compared. The clinical significance of miR-28-5p in colon cancer and the association with the survival were determined. The predictive value of miR-28-5p in clinical features was determined using receiver operating characteristic curve. The target genes of miR-28-5p were identified, and the functional of target genes was performed using bioinformatics analysis.

**Results:**

: The expression of miR-28-5p was increased in colon cancer tissues compared with normal controls (*p* = 0.037). The expression of miR-28-5p was significantly increased in tissues with distant metastases compared with that without distant metastases (*p* = 0.026). Patients with high expression of miR-28-5p have a shorter survival time than those with low expression (*p* = 0.004). Cox analysis showed that miR-28-5p was an independent predictor for the survival of patients (*p* = 0.014). Combination of miR-28-5p with TNM stage and clinical stage can improve the prognostic value for the patients (*p* < 0.05). miR-28-5p has a moderate predictive value in predicting the TNM stage and clinical stage (T stage: AUC = 0.515; N stage: AUC = 0.523, M stage: AUC = 0.572; clinical stage: AUC = 0.539). 711 potential target genes of miR-28-5p were screened; their function and pathways were identified.

**Conclusions:**

: This study demonstrated that miR-28-5p was increased in colon cancer and can be an independent indicator for the overall survival in patients with colon cancer.

## 1. Introduction

Colorectal cancer (CRC) is one of the leading malignant cancers in the world, ranking third for incidence (10.2%, with 1.8 million new cases) and second for mortality (9.2%, with 881,000 deaths) of all cancers [1, 2]. Colon cancer is a common subtype of CRC, and the survival of patients with colon cancer is varied greatly between early and advanced stage [3]. Thus, early detection and prediction the prognosis of colon cancer is crucial for the treatment in patients with colon cancer. Previous studies have reported that some biomarkers, including genes, miRNAs, were capable of early detecting and predicting the prognosis in patients with colon cancer [4, 5]. However, these results were often based on single center with few patients, which undermine the robustness of the results. Therefore, finding reliable biomarkers that could be used to early detect and predict the prognosis of colon cancer remains an urgent need for the doctors.

miRNAs are noncoding RNA molecules of 21-24 nucleotides that regulate the expression of target genes in a post-transcriptional manner. They have been implicated in many cancers and associated with clinical features, such as disease stage and survival in patients [6]. To date, some miRNAs were found to be valuable indicators for the prognosis for colon cancer. For example, miR-506 could greatly differentiate early-stage CRC from healthy individuals with 76.8% specificity and 60.7% sensitivity [7]. High miR-34a levels in CRC predict a rather increased risk for disease recurrence and poor overall survival, particularly in patients at an early TNM stage [8].

miR-28-5p has been implicated in several cancers, such as ovarian cancer [9], prostate cancer [10], and hepatocellular carcinoma [11]. Expression of miR-28-5p also found to be decreased in liver metastases tissues compared with the primary colorectal cancer tissues [12, 13]. More recently, Tsiakanikas et al. [14] report that miR-28-5p was downregulated in CRC compared with their adjacent noncancerous mucosae, suggesting that miR-28-5p was involving in the development of CRC. However, the current knowledge of miR-28-5p in colon cancer is limited. Thus, this study aimed to determine the clinical significance and potential prognostic value of miR-28-5p in colon cancer by using the data from The Cancer Genome Atlas (TCGA) and Gene Expression Omnibus (GEO) dataset and our colon cancer tissues.

## 2. Materials and Methods

### 2.1. Expression of miR-28-5p in Colon Cancer from TCGA and GEO Database

In order to determine the association between miRNAs and the colon cancer, the data of colon adenocarcinoma (COAD) was downloaded from the TCGA database (https://cancergenome.nih.gov/). We also downloaded a colon cancer dataset (GSE49246) [15] from the GEO database. The COAD dataset from TCGA includes miRNA expression data from 453 colon cancer tissues and eight normal colon tissues. The GSE49246 dataset includes 40 colon cancer tissues and 40 adjacent normal tissues. The expression of miR-28-5p among gastrointestinal cancers, including COAD, esophageal cancer (ESCA), liver hepatocellular carcinoma (LIHC), rectal adenocarcinoma (READ), cholangiocarcinoma (CHOL), gastric cancer (STAD), was analyzed using OncomiR website online tool (http://www.oncomir.org), which uses the data from TCGA database.

### 2.2. Tissue Samples Collection

Forty colon cancer tissues and corresponding adjacent normal tissues were collected from the Biobank of Guangxi Medical University Cancer Hospital (Nanning, China) between January 2015 and December 2017. Fresh tissue samples were frozen within 30 min after surgery and stored in liquid nitrogen until use. The inclusion criteria were that the tissue histologically proven colon cancer, no severe major organ dysfunction, no prior cancer chemotherapy. This study was approved by the Ethics Committee of Guangxi Medical University Cancer Hospital; written informed consent was obtained from each patient.

### 2.3. RNA Isolation from Colon Cancer Tissues

Total RNA from colon cancer and adjacent normal tissues was isolated using TRIzol reagent (Invitrogen; Thermo Fisher Scientific, Inc., Waltham, MA, USA) according to the manufacturer's protocol. RNA concentration was measured using NanoDrop ND-1000 (Thermo Fisher Scientific, Inc.), and the quality was assessed using electrophoresis with 1.5% denaturing agarose gels. TaqMan probe-based qPCR was performed using a commercial kit (Applied Biosystems; Thermo Fisher Scientific, Inc.) according to the manufacturer's protocol.

### 2.4. qRT-PCR Procedure

The qRT-PCR procedure was performed using a miR-28-5p specific primer and ABI's TaqMan MicroRNA Reverse Transcription kit (Applied Biosystems; Thermo Fisher Scientific, Inc.). U6 was used as the internal control. The following primers were used: miR-28-5p forward, 5′-GGT AAG TCA CGCGGT-3′ and reverse, 5′-CAG TGC GTC TCG TGG AGT-3′; U6 forward, 5′-CTC GCT TCG GCA GCA CA-3′ and reverse, 5′-AAC GCT TCA CGA ATT TGC GT-3′. The reaction conditions included 1 cycle at 95°C for 5 minutes; 15 cycles at 95°C for 25 seconds, 64°C for 20 seconds, 72°C for 20 seconds; and a final 31 cycles at 93°C for 25 seconds, 64°C for 20 seconds, 72°C for 20 seconds. Amplicons were detected using capillary electrophoresis on an ABI 3130xl Genetic Analyzer (Applied Biosystems/Life Technologies, Grand Island, NY). miR-28-5p levels were quantified using the 2 − ΔΔCq method.

### 2.5. Analysis of miR-28-5p in Colon Cancer

The expression of miR-28-5p was firstly compared among gastrointestinal cancers using the data from the TCGA database, including COAD, ESCA, LIHC, READ, CHOL, and STAD. Then, the association of miR-28-5p with clinical features was analyzed using the data from TCGA, GEO database, and our center. Next, the prognostic value of miR-28-5p in patients with colon cancer was analyzed, and the jointed effect of the combination of miR-28-5p with clinical features was performed. Finally, the predictive value of miR-28-5p on clinical features was analyzed.

### 2.6. miR-28-5p Target Genes Prediction and Functional Analysis

To explore the role of miR-28-5p in the diseases and the possible mechanism, we examined the function of miR-28-5p target genes and the pathway they involve. The target genes of miR-28-5p were predicted using the mirDIP database (http://ophid.utoronto.ca/mirDIP/), which integrated 15 miRNA prediction databases [16]. Then, the function of target genes was analyzed using Gene Ontology (GO) analysis, including molecular function (MF), biological process (BP) and cellular component (CC), and the Kyoto Encyclopedia of Genes and Genomes (KEGG) pathway was also analyzed using clusterProfiler package [17] running in *R* language (version 3.5.2).

### 2.7. Statistical Analysis

Data are presented as the mean ± standard deviation. The *χ*2 was used to compare the differences of categorical variables and the Student's *t* test was used for comparison of differences between two groups. Kaplan-Meier survival curves and the log-rank test were used to analyze the survival rate in patients with colon cancer. Multivariate Cox proportional hazards regression models were performed to explore the prognostic value of multiple variables in colon cancer patients. The predictive value of miR-28-5p in clinical features was using the receiver operating characteristic (ROC) curve. All statistical analyses were performed using the *R* language (version 3.4.1). *p* < 0.05 was considered to indicate a statistically significant difference.

## 3. Results

### 3.1. Expression of miR-28-5p in Gastrointestinal Cancers

**Figure 1:**
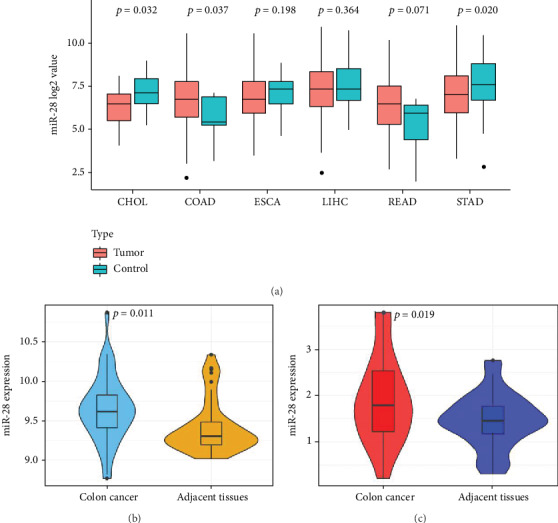
(a) Expression of miR-28-5p in gastrointestinal cancers. (b) Comparison of miR-28-5p in colon cancer and adjacent normal tissues from GSE49246. (c) Comparison of miR-28-5p in colon cancer and adjacent normal tissues from our center.

Table 1 listed the baseline data in patients with colon cancer. All the histopathological types of colon cancer were colon adenocarcinoma in three datasets, and the stage of COAD was assessed based on the American Joint Committee on Cancer criteria, 8th Edition. By analyzing the expression value of miR-28-5p in gastrointestinal cancer using the TCGA dataset, we found that the expression of miR-28-5p was significantly increased in COAD compared with corresponding normal tissues (*p* = 0.037), but we failed to find significant differences in ESCA (*p* = 0.198), LIHC (*p* = 0.364), and READ (*p* = 0.071), while expression of miR-28-5p was decreased in CHOL and STAD compared with the corresponding normal tissues (*p* = 0.032). The data from GSE49246 and our clinical colon cancer tissues also revealed that miR-28-5p was increased in colon cancer tissues compared with adjacent normal tissues (GSE49246: *p* = 0.011; our data: *p* = 0.019). Figures 1(b) and 1(c). These results suggesting that miR-28-5p was increased in colon cancer.

### 3.2. The Association of miR-28-5p with Clinical Features

As shown in Table 2, the expression of miR-28-5p in the TCGA COAD dataset was significant difference regarding the patients' age and M stage (*p* < 0.05), and the expression of miR-28-5p in elder patients and patients at M1 stage was higher than in young patients and patients at M0 stage. However, a not significant difference was found regarding the T stage, N stage, and clinical stage (*p* > 0.05). The data of GSE49246 showed that the expression of miR-28-5p has no obvious difference in the patient's gender and age (*p* > 0.05). The similar results were observed in our data, and our results also found that the expression of miR-28-5p was remarkably increased in cancer at advance clinical stage (III+IV) compared with early clinical stage (I+II), with the *p* value as 0.005, indicating that miR-28-5p was closely related to the distant metastasis of colon cancer.

### 3.3. Prognostic Value of miR-28-5p in Patients with Colon Cancer

**Figure 2:**
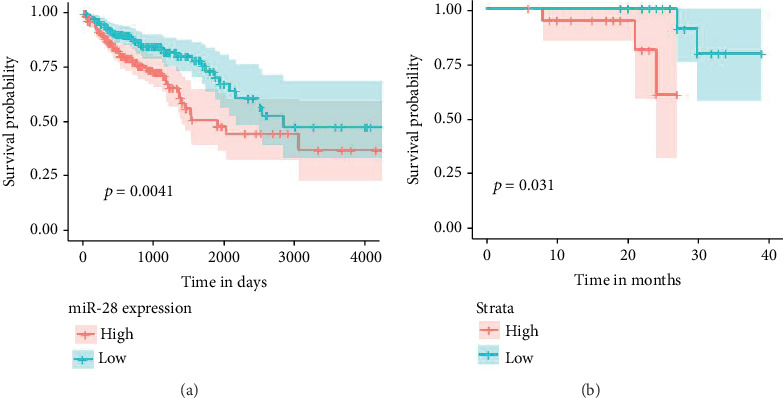
(a) Survival analysis of miR-28-5p in colon cancer patients using TCGA data. (b) Survival analysis of miR-28-5p in colon cancer patients using clinical tissue data.

The results of the TCGA dataset showed that high expression of miR-28-5p indicated a shorter survival in patients with colon cancer; with the log-rank *p* values as 0.004, this result was confirmed by our data, and the log-rank values were 0.031. The multivariant Cox regression analysis for the TCGA and our data further revealed that miR-28-5p was an independent factor for the prognosis in patients with colon cancer (TCGA dataset: *p* = 0.014; our dataset: *p* = 0.049), suggesting that miR-28-5p was a good prognostic indicator for the overall survival in patients with colon cancer. See Figure 2.

### 3.4. Joints Effect of Combination of miR-28-5p with Clinical Features

**Figure 3:**
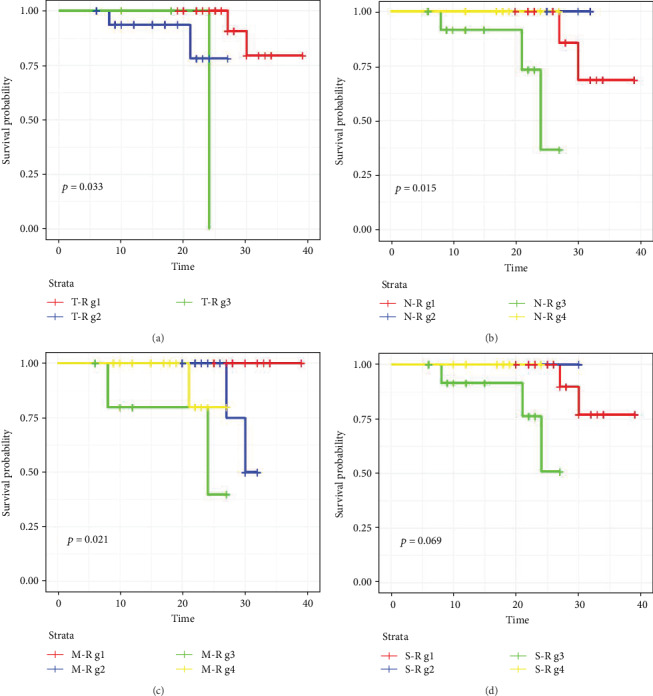
Kaplan-Meier survival curves for the combination of miR-28-5p with clinical features. (a) Combination of miR-28 with T stage. T-R g1: miR28-high+T stage-low; T-R g2: miR28-high+T stage-high; T-R g3: miR28-low+T stage-high; T-R g4: miR28-low+T stage-low. (b) Combination of miR-28 with N stage. N-R g1: miR28-high+N stage-low; N-R g2: miR28-high+N stage-high; N-R g3: miR28-low+N stage-high; N-R g4: miR28-low+N stage-low. (c) Combination of miR-28 with M stage. M-R g1: miR28-high+M stage-low; M-R g2: miR28-high+M stage-high; M-R g3: miR28-low+M stage-high; M-R g4: miR28-low+M stage-low. (d) Combination of miR-28 with clinical stage. S-R g1: miR28-high+clinical stage-low; S-R g2: miR28-high+clinical stage-high; S-R g3: miR28-low+clinical stage-high; S-R g4: miR28-low+clinical stage-low.

In order to identify more indicators that predicting the prognosis of colon cancer, we combined the expression of miR-28-5p with TNM stage and clinical stage using the data from TCGA and found that different combinations of miR-28-5p with TNM stage and clinical stage were associated with the survival in patients with colon cancer (Figure 3). The multivariate Cox regression also revealed that these combinations could be acted as independent prognostic indicators in patients with colon cancer (*p* < 0.05) (Table 3).

### 3.5. Predictive Value of miR-28-5p on Clinical Features

**Figure 4:**
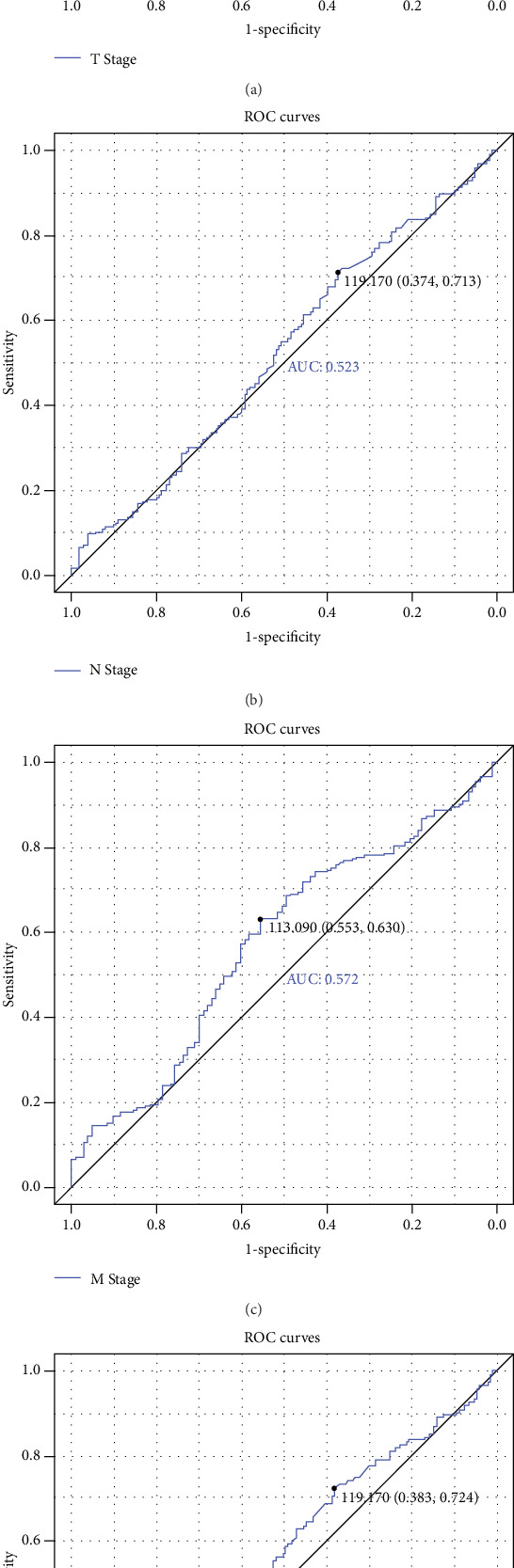
Predictive value of miR-28-5p on clinical features. (a) T stage. (b) N stage. (c) M stage. (d) Clinical stage.

Due to the small sample size of our data, we used the data from TCGA to examine the relationship between the miR-28-5p and clinical features. We divided the clinical features into binary variable and tested the predictive value of miR-28-5p using ROC analysis. As shown in Figures 4(a)–4(d), the predictive value of the miR-28-5p on these clinical features was moderated, with T stage (AUC = 0.515), N stage (AUC = 0.523), M stage (AUC = 0.572), and clinical stage (AUC = 0.539), respectively.

### 3.6. Functional Analysis of miR-28-5p Target Genes

**Figure 5:**
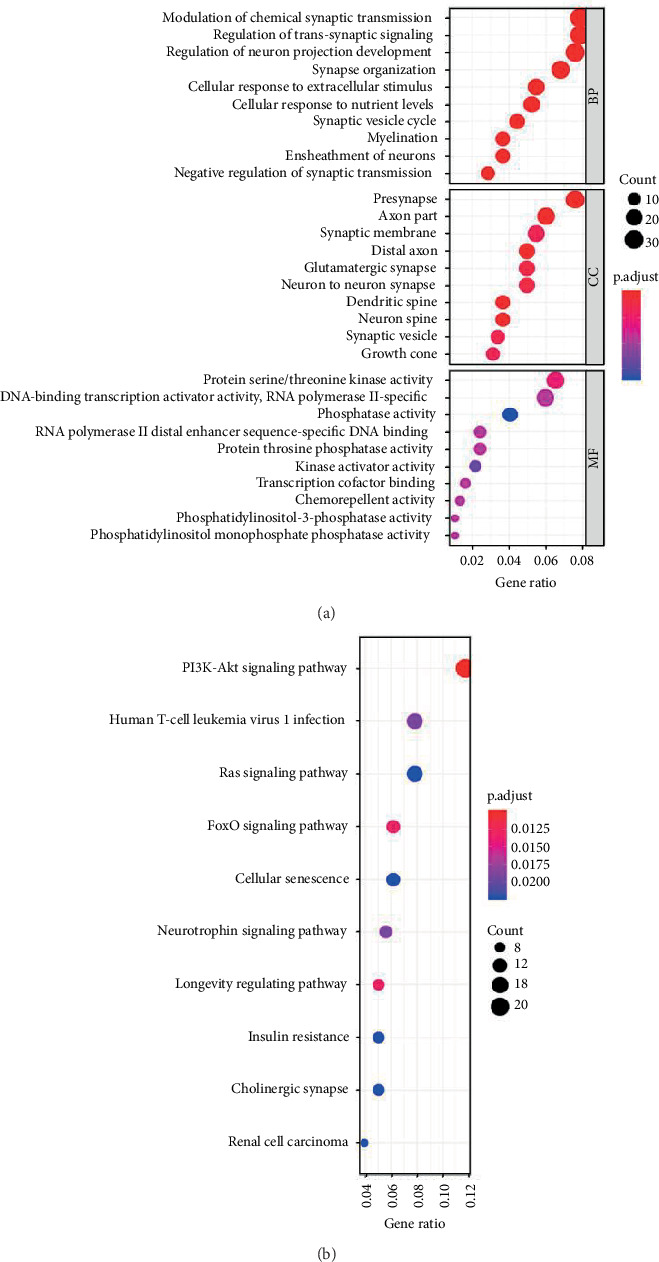
Functional analyses for the target genes of miR-28-5p. (a) Gene Ontology analysis for the target genes. (b) KEGG analysis for the target genes.

The mirDIP database identified 711 target genes of miR-28-5p (Supplementary Materials (available here)); we selected the first 400 target genes for the functional analysis. The GO analysis showed that the most enrichment of BP is the modulation of chemical synaptic transmission, and the CC is presynapse; MF is protein serine/threonine kinase activity. The most enrichment of the KEGG pathway is the PI3K-Akt signaling pathway (Figure 5).

## 4. Discussion

There are several advantages of using miRNAs as prognostic biomarkers in cancers compared with mRNA and protein. First, the number of candidate miRNAs is much smaller than the protein-coding mRNA. Second, miRNAs are highly stable, especially in formalin-fixed, paraffin-embedded tissues compare with mRNAs. Third, qRT-PCR method is high sensitivity, cost-efficient in the detection of miRNAs. The limitations including that the samples must pass quality controls to avoid high degradation of nucleic acids or contamination of other miRNAs released by other cells or tissues [18].

miR-28-5p is an intragenic miRNA, which has been reported to be downregulated expressed in several tumor types, such as hepatocellular carcinoma [19] and renal cell carcinoma [20], but also upregulated in some other cancers, such as ovarian, esophageal, and cervical cancer [9, 21, 22]. With regard to the colon cancer, the expression of miR-28-5p was inconsistent in previous studies [14, 15]. The reason of the inconsistent results can be attributed to the different sample or the detected methods, or different sample types, such as tissue specimen or serum sample. In this study, we determined the expression of miR-28-5p in colon cancer tissues using data from TCGA dataset, GEO dataset, respectively, which is using RNA sequencing or microarray technique to test the expression of miR-28-5p, and we also determined the expression of miR-28-5p in tumor tissues using the qRT-PCR method and performed functional analysis for the target genes of miR-28-5p, which provided a more reliable result compared with previous studies.

Previous studies have indicated that miR-28-5p regulated cell proliferation, migration, invasion, and in CRC [23, 24]. In this study, by analyzing the data from TCGA, GEO dataset, and our clinical specimens, we found that the expression of miR-28-5p was increased in colon cancer tissues compared with corresponding normal tissues, suggesting that miR-28-5p may act as an oncogene during the process of colon carcinogenesis. This result is contrary to the Tsiakanikas et al. [14] report, who analyzed the expression of miR-28-5p in 182 CRC and 86 paired noncancerous colorectal mucosae. In addition, although no obvious difference among different T stage, N stage, we found that miR-28-5p was significantly increased in colon cancer in the M1 stage compared with the M0 stage, indicating that expression of miR-28-5p was associated with the progression of colon cancer.

Patient at advance TNM stage or clinical stage is likely to have a poor prognosis. Therefore, finding biomarkers that can detect colon cancer before advance TNM stage or clinical stage could increase the chances of early intervention and improve patient's survival. Currently, many miRNAs have been shown to be useful biomarkers for the early detection of CRC [25–27]. In a previous report [13], miR-28-5p in primary colon cancer tissues was showed to downregulate compared with the tissues of liver metastases. In a recent study, Wang et al. [28] reported that serum miR-28-5p expressions were correlated with the TNM stage and liver metastasis. In this study, both our data and the TCGA data showed that miR-28-5p was associated with distant metastasis of colon cancer, although no association was found in lymph node metastasis, suggesting that high expression of miR-28-5p indicating a high chance of tumor distant metastasis of colon cancer.

Molecular biomarkers serve an important role in the therapeutic decision-making process, as they can be indicators for the patients to receive individual chemotherapeutic interventions. Although miR-28-5p has been reported to be aberrantly expressed in various human cancer [8, 9, 11]. However, limited knowledge is available about the association between miR-28-5p expression and the survival in patients with colon cancer. In the present study, we analyzed the prognostic value of miR-28-5p for patients with colon cancer and found that colon cancer patients with high expression of miR-28-5p were associated with a poor survival. Further analysis using multivariate Cox analysis also indicated that miR-28-5p was an independent indicator for the prognosis of colon cancer patients. These results were in agreement with Tsiakanikas et al. [14] report, which showed that high expression of miR-28-5p indicated poor disease-free survival and overall survival of CRC patients.

Since the above results showed that miR-28-5p was associated with the metastasis of colon cancer, we further examined the predictive value of miR-28-5p in the clinical features using ROC analysis. However, the results show the predictive value was moderate, including TNM stage and clinical stage, suggesting that we could not use miR-28-5p to clearly divide the patients into early or advanced TNM stage and clinical stage. Likewise, we also explored the combinations of miR-28-5p with the clinical features; by combining the miR-28-5p expression with TNM stage and clinical stage, we found that these combinations could clearly divide the patients into four groups and could be used as independent predictors to the survival in patients with colon cancers, suggesting that these combinations could significantly increase the prognostic value for patients with colon cancers.

Regarding the mechanism of miR-28-5p in the regulation of cancer cells, previous studies observed that miR-28-5p inhibited CAMTA2 expression and regulates colon cancer progression by suppressing Wnt/*β*-catenin signaling [29]. As a direct target gene of miR-28-5p, SSRP1 promotes CRC progression and is negatively regulated by miR-28-5p [24]. miR-28-5p also reduced CRC cell proliferation, migration, and invasion in vitro by inhibiting CCND1 expression [30]. In the present study, we identified the potential target genes of miR-28-5p and revealed the function of these target genes through bioinformatics analysis; these results were helpful to uncover the possible mechanism of miR-28-5p in diseases.

Compared with Tsiakanikas et al. [14] study, our study only focus the colon cancer, and the results were validated by other datasets, which provided a more reliable results than previous studies. However, some limitations need to be noted. First, the sample size of our clinical colon cancer tissues was small; a larger number of samples was necessary to verify these results. Second, colon cancer is characteristic by microsatellite instability and chromosome instability, but our study did not analyze the impact of these factors. Third, some important gene mutation, such p53, K-ras mutation frequently occurs in the colon cancer, but we did not analyze the association between miR-28-5p and these genes. Fourth, although we conducted a functional analysis for the target genes of miR-28-5p using bioinformatics methods, the biological function of miR-28-5p needs to explore by using *in vivo* and *in vitro* experiments. Therefore, further study is needed by taking these factors into account in order to confirm the prognostic and early predict value of miR-28-5p in colon cancer.

## 5. Conclusion

The present study demonstrates that the expression miR-28-5p is increased in colon cancer and associated with the distant metastasis of cancer. High expression of miR-28-5p indicates a poor prognosis of colon cancer patients, which might serve as an independent indicator for the prognosis.

## Figures and Tables

**Table 1:** Characteristic of the patients with colon cancer in this study.

	TCGA data	GEO data	Our date
Age	69 (31-98)	65 (32-83)	57 (31-84)
Gender			
Male/female	269/244	21/19	22/18
Differentiation			
Low/moderate/high			8/28/4
Location			
Right/left site	284/229		18/22
T stage			
T1/T2/T3/T4	9/85/355/63		0/3/12/25
N stage			
N0/N1/N2	298/121/84		13/14/13
M stage			
M0/M1/MX	381/77/55		24/16/0
Clinical stage			
I/II/III/IV	84/202/150/77		1/7/14/18

**Table 2:** Association of miR-28-5p with clinical parameters in colon cancer patients.

	TCGA	*p* value	GSE49246	*p* value	Our data	*p* value
Age						
>60 years	105.3 ± 30.0	0.004	9.6 ± 0.5	0.621	2.21 ± 0.87	0.048
≤60 years	115.1 ± 33.7		9.6 ± 0.3		1.63 ± 0.92	
Gender						
Male	106.4 ± 30.9	0.170	9.6 ± 0.3	0.827	1.72 ± 1.06	0.138
Female	110.6 ± 32.0		9.7 ± 0.5		2.09 ± 0.72	
Location						
Right site	104.44 ± 31.11	0.048			1.77 ± 0.87	0.213
Left site	110.08 ± 32.84				2.07 ± 1.36	
T stage						
T1+T2	108.8 ± 33.7	0.89			1.66 ± 0.80	0.199
T3+T4	108.3 ± 30.9				2.14 ± 0.46	
N stage						
N0	107.0 ± 30.9	0.276			1.61 ± 0.73	0.152
N1+N2	110.4 ± 32.2				2.06 ± 1.05	
M stage						
M0	106.4 ± 31.6	0.026			1.47 ± 0.56	0.004
M1+MX	114.1 ± 30.3				2.16 ± 1.04	
Clinical stage						
I+II	110.8 ± 31.8	0.161			1.55 ± 0.68	0.005
III+IV	106.4 ± 31.6				2.83 ± 0.92	

**Table 3:** Multivariate Cox analysis for the combination of miR-28-58 and clinical features in colon cancer patients.

	HR	95% CI	*p* value
miR-28/high+T/low	—	—	—
miR-28/high+T/high	2.25	0.69-7.31	0.178
miR-28/low+T/low	0.35	0.03-3.38	0.366
miR-28/low+T/high	4.11	1.28-13.17	0.017
miR-28/high+N/low	—	—	—
miR-28/high+N/high	1.52	0.81-2.86	0.187
miR-28/low+N/low	0.72	0.36-1.42	0.348
miR-28/low+N/high	3.27	1.85-5.78	<0.001
miR-28/high+M/low	—	—	—
miR-28/high+M/high	1.73	0.87-3.47	0.116
miR-28/low+M/low	0.72	0.41-1.26	0.252
miR-28/low+M/high	3.36	1.96-5.76	<0.001
miR-28/high+stage/low	—	—	—
miR-28/high+stage/high	2.02	1.01-4.05	0.045
miR-28/low+stage/low	0.97	0.46-2.02	0.94
miR-28/low+stage/high	4.77	2.55-8.92	<0.001

## Data Availability

Answer: Yes. Comment: The data used to support the findings of this study are included within the article. (Table 1).

## Abbreviations

CRC: Colorectal cancer
TCGA: The Cancer Genome Atlas
GEO: Gene Expression Omnibus
OS: Overall survival
ROC: Receiver operating characteristic
CAMTA2: Calmodulin binding transcription activator 2
CCND1: Cyclin D1
qRT-PCR: Quantitative real-time polymerase chain reaction.

## Supplementary Materials

Supplementary Materials The mirDIP database identified the target genes of miR-28-5p.
